# Stunting coexisting with overweight in 2·0–4·9-year-old Indonesian children: prevalence, trends and associated risk factors from repeated cross-sectional surveys

**DOI:** 10.1017/S1368980016000926

**Published:** 2016-04-28

**Authors:** Cut Novianti Rachmi, Kingsley Emwinyore Agho, Mu Li, Louise Alison Baur

**Affiliations:** 1 Discipline of Paediatrics and Child Health, The Children’s Hospital at Westmead (University of Sydney Clinical School), Locked Bag 4001, Westmead, NSW 2145, Australia; 2 School of Science and Health, University of Western Sydney, Penrith, NSW, Australia; 3 Sydney School of Public Health, The University of Sydney, Sydney, NSW, Australia

**Keywords:** Stunting, Overweight/obesity, Indonesia, Children, Double burden

## Abstract

**Objective:**

The persistence of undernutrition, along with overweight and obesity, constitute the double burden of malnutrition. The present study aimed to: (i) describe the prevalence and trends of concurrent stunting and overweight in Indonesian children; (ii) identify potentially associated risk factors; and (iii) determine whether stunted children are at greater risk of overweight compared with those of healthy height.

**Design:**

A secondary data analysis of children aged 2·0–4·9 years in four cross-sectional studies of the Indonesian Family Life Survey. Children’s height and BMI *Z*-scores were calculated based on the WHO Child Growth Standards (2006). We defined ‘concurrent stunting and overweight’ as height-for-age *Z*-score <−2 and BMI *Z*-score >+1. Multivariate generalised linear latent and mixed models were used to determine associated risk factors.

**Setting:**

Thirteen out of twenty-seven provinces in Indonesia.

**Subjects:**

Children (*n* 4101) from four waves of the Indonesian Family Life Survey (1993–2007).

**Results:**

There were inconsistent trends in the prevalence of concurrent stunting and overweight from waves 1 to 4. Children were more likely to be stunted and overweight when they were in the youngest age group (2·0–2·9 years), were weaned after the age of 6 months, had short-statured mothers or lived in rural areas. Stunted children were significantly more likely to be overweight than healthy-height children (OR>1) but did not differ significantly different across each wave (OR=1·34–2·01).

**Conclusions:**

Concurrent stunting and overweight occurs in Indonesian children aged 2·0–4·9 years. Current policies and programmes need to be tailored for the management of this phenomenon.

The ‘double burden of malnutrition’ – the persistence of undernutrition, along with a rapid increase in overweight and obesity – is now recognised as ‘the new normal’^(^ ^1^ ^,^ ^2^ ^)^. Many countries face the double burden of malnutrition, although it is a more common phenomenon in countries where stunting rates are high^(^ ^1^ ^)^. Several studies and reviews have also shown that this phenomenon can be found at the level of both the family (mother and child double burden)^(^ ^3^ ^–^ ^8^ ^)^ and the individual^(^ ^4^ ^,^ ^9^ ^,^ ^10^ ^)^.

The co-occurrence of stunting and overweight has been described in children from such countries as Mexico, China, Russia, South Africa, Brazil and the USA^(^ ^9^ ^–^ ^12^ ^)^. A few studies – from Uruguay, Ecuador, Guatemala, South Africa and Mexico – have reported the phenomenon in children aged less than 5 years^(^ ^4^ ^,^ ^13^ ^–^ ^16^ ^)^. However, to our knowledge, no study has reported on concurrent stunting and overweight prevalence, or the associated risk factors, in any paediatric age group in South-East Asia. A better understanding of the risk factors related to concurrent stunting and overweight would improve prevention and management approaches aimed at overcoming this problem.

The present paper is the second of a series of secondary data analyses on the double burden of malnutrition in Indonesia. In our first paper we showed that, over a 14-year time frame (1993 to 2007), the prevalence of stunting in Indonesian children aged 2·0–4·9 years decreased by 14·1% from 50·8% to 36·7%, while the prevalence of overweight increased by 6·2% from 10·3% to 16·5% (all *P*<0·01). We also identified that associated risk factors for a higher probability of being stunted or underweight included lower birth weight (<2·5 kg), being breast-fed for 6 months or more, having a mother or father who was underweight or short-statured, and mothers with no formal education. The likelihood of being stunted was also higher when a child lived in a rural area (all *P*<0·05). Children were more likely to be at risk or overweight/obese if they were in the youngest age group (2·0–2·9 years), male, had parents who were overweight/obese and fathers with high formal education (university or more; all *P*<0·05)^(^ ^17^ ^)^.

Herein we elaborate further on the co-occurrence of stunting and at risk of or overweight/obesity in the same individual – we refer to this as ‘concurrent stunting and overweight’. The aims of the current paper were to: (i) describe the prevalence and trends of concurrent stunting and overweight in young Indonesian children between 1993 and 2007; (ii) identify potential risk factors associated with the phenomenon; and (iii) determine whether stunted children are at greater risk of being overweight or obese compared with their healthy-height peers.

## Methods

### Indonesian Family Life Survey

#### Data collection

Data were from the first four waves of the Indonesian Family Life Survey (IFLS) in the years 1993, 1997, 2000 and 2007^(^ ^18^ ^)^. Details of the IFLS have been described in our first paper and several previously published field reports^(^ ^17^ ^,^ ^19^ ^,^ ^20^ ^)^. In brief, IFLS is a longitudinal, nationally representative survey of a stratified random sample of households involving both questionnaires and anthropometric measurements. The first wave (1993) recruited participants from thirteen of the twenty-seven Indonesian provinces. The next three surveys followed the same survey and measurement methods as the first one, and had a very high re-contact rate (>90%). Trained professionals collected the data. The original design of the survey was longitudinal, targeting the same families and the children of the families from the first wave of the survey^(^ ^18^ ^–^ ^20^ ^)^. In the present paper we use cross-sectional study design. We analysed data from children aged 2·0–4·9 years in each wave, resulting in different groups of children in each of the four waves.

#### Inclusion criteria

Inclusion criteria were children aged 2·0–4·9 years who had complete records for child information (height, weight, age and sex) and matching parental-, household- and community-level data. The minimum age was chosen at 2 years because the process of stunting is more prominent before the age of 2^(^ ^21^ ^)^.

#### Ethics

Ethics approval was granted from the Institutional Review Board at Rand Corporation (USA) and from the Ethics Committee at Universitas Indonesia (Indonesia) for the first wave and the Ethics Committee at Universitas Gadjah Mada (Indonesia) for the next three waves.

#### Anthropometric indices calculations

BMI was calculated as weight/height^2^ (kg/m^2^). Using Pan and Cole’s LMS Growth Program^(^ ^22^ ^)^, the children’s height and BMI *Z*-scores were calculated based upon the WHO Child Growth Standards (2006)^(^ ^23^ ^)^. Stunting was defined as height-for-age *Z*-score <−2. Children with BMI *Z*-score >+1, >+2 and >+3 were categorised as being at risk of overweight, overweight and obese, respectively^(^ ^23^ ^,^ ^24^ ^)^. For the purposes of the present study, we defined ‘concurrent stunting and overweight’ as children with the combination of height-for-age *Z*-score <−2 and BMI *Z*-score >+1.

### Potentially associated risk factors

The conceptual framework for the current analysis was modified from the ecological model of childhood obesity of Davison and Birch^(^ ^25^ ^)^ to include not only the available variables in the IFLS data set, but also stunting as a form of malnutrition. The potential risk factors were divided into three categories: child-, parental- and household-, and community-level factors.

#### Child-level factors

These consisted of the child’s age, sex, birth weight (for whom this had been recorded), whether they were ever breast-fed, age of weaning (defined as full cessation of breast-feeding), age of starting complementary foods, and their current weight and height. Age was divided into three groups: 2·0–2·9, 3·0–3·9 and 4·0–4·9 years. Birth weight was categorised as low birth weight (<2·5 kg), healthy birth weight (2·5–<4·0 kg) and high birth weight (≥4·0 kg). Both age of weaning and age of starting complementary foods were divided into two groups: <6 months and ≥6 months.

#### Parental- and household-level factors

Parental-level factors included maternal and paternal factors. Maternal factors included mothers’ age, BMI, height and maternal history of check-up during pregnancy. Maternal age was categorised as <30 or ≥30 years and maternal BMI was categorised based on the WHO BMI International Classification cut-off points of ≥25 and ≥30 kg/m^2^ for overweight and obesity, respectively^(^ ^26^ ^)^. Because of the lack of consistent definitions of stunting for men and women in the literature, for the purposes of the current analysis we categorised height as short stature (height-for-age *Z*-score <−2) or healthy height (height-for-age *Z*-score ≥−2), based upon a standard age of 19 years and the WHO Standard Growth Reference for School-Aged Children and Adolescents^(^ ^27^ ^)^. Maternal history of check-up during pregnancy was categorised as ever or never had check-up (yes/no variable). Paternal factors included fathers’ age, BMI and height using the same cut-off points as mothers, and parental marital status.

The household-level factors included mothers’ and fathers’ education (divided into four groups: never attended any formal education, attended primary school, middle school, and university or higher) and the household’s wealth index, assessed by calculation of a score involving the ownership of eleven household assets by using weights. We ranked the households into five quintiles: poorest, poorer, average, richer and richest. For the analysis, households in the bottom two quintiles were categorised as poor, those in the middle two quintiles as average, and those in the highest quintile as rich households^(^ ^28^ ^)^.

#### Community-level factors

The community factors included the housing area (rural and urban) and region. Four regions were included in the study: Sumatra, Java, Bali and Nusa Tenggara Barat, and Kalimantan and Sulawesi.

### Statistical analysis

In the IFLS, each household completed several separate questionnaires, each with different types of information (e.g. anthropometry, household economy, child information, adult information). These different files were merged in order to build the data set for analysis. We used sampling weights in the analysis to reduce bias; however, we did not adjust sampling weight for children aged 2·0–4·9 years because sub-samples are mutually exclusive. Frequency tabulations were first conducted to describe the distributions of data used in the study, followed by prevalence estimates using the Taylor-series linearisation method to examine the impact of all potential predictors using *χ* ^2^ tests and multiple testing with the Bonferroni correction was carried out by dividing the 5% significance level by the number of *χ* ^2^ tests performed.

The unadjusted odd ratios for factors associated with stunting and overweight were examined using GLLAMM (generalised linear latent and mixed models)^(^ ^29^ ^)^. This was followed by multivariable analyses after controlling for community-, child-, parental- and household-level factors. All statistical analyses were conducted using the statistical software package STATA/MP version 13.1 (2014) and multilevel models were fitted using STATA commands to adjust for the variability of clustering.

In the multivariable analysis models, a manual stepwise backward elimination process was used to identify factors that were significantly associated with the study outcome using a 5% significance level. In order to minimise or avoid statistical error in our analyses, we repeated the backward elimination process using a different approach. First, only variables among community-, child-, parental- and household-level variables with *P* < 0·20 identified in the univariate analysis were entered for the backward elimination process. Second, we double-checked the backward elimination by including all community, child, parental and household variables, and only the variables with *P*<0·05 were retained in the final model (i.e. child’s age group, age of weaning, maternal height and housing area). Third, we tested and reported any collinearity in the final model. The odd ratios and 95% confidence intervals were calculated for each variable and were used to measure the impact of the adjusted estimates on the study outcome. The significant Bonferroni-adjusted *P* values are reported.

The odds ratio of becoming overweight for those who were stunted was calculated by dividing the probability of being overweight in stunted children by the probability of being overweight in the healthy-height children.

## Results

### Characteristics of participants

The sociodemographic characteristics of the participants and their parents are shown in Table 1. There were a total of 4101 children aged 2·0–4·9 years in all four waves, with a similar percentage of children in each age band and sex. In all four waves most children were born in the healthy weight range (2·5–4·0 kg) and were breast-fed until 6 months. Throughout all four waves, a little more than half of the mothers were aged ≥30 years or were classified as having short stature, except in wave 4 where 54·5% of mothers were of healthy height. As many as 83% of all fathers were aged ≥30 years during the data collection and just over half (54%) of fathers were of short stature. The prevalence of underweight in both mothers and fathers remained relatively constant throughout the four waves, while the prevalence of overweight in both mothers and fathers increased over time. At the household level, there were more educated fathers than mothers. From years 1993 to 2000, there were more people living in rural areas, but by year 2007 more families were living in urban areas.

**Table 1:** Characteristics of children and parents in each wave (wave 1, 1993; wave 2, 1997; wave 3, 2000; wave 4, 2007) of the Indonesian Family Life Survey

	Wave 1 (*n* 938)		Wave 2 (*n* 913)		Wave 3 (*n* 939)		Wave 4 (*n* 1311)		All waves (*n* 4101)	
Characteristic	*n* or Mean	% or sd	*n* or Mean	% or sd	*n* or Mean	% or sd	*n* or Mean	% or sd	*n* or Mean	% or sd
Child level										
Age										
2·0–2·9 years	313	33·4	274	30·0	299	31·8	425	32·4	1311	32·0
3·0–3·9 years	326	34·8	257	28·2	294	31·3	454	34·6	1331	32·5
4·0–4·9 years	299	31·8	382	41·8	346	36·9	432	33·0	1459	35·5
Sex										
Male	501	53·4	462	50·6	482	51·3	633	48·3	2078	50·7
Female	437	46·6	451	49·4	457	48·7	678	51·7	2023	49·3
Birth weight (*n* 2420)										
<2·5 kg	46	7·8	27	6·1	19	7·7	75	6·6	167	6·9
2·5–<4·0 kg	473	80·6	357	81·1	198	79·8	963	84·1	1991	82·2
≥4·0 kg	68	11·6	56	12·8	31	12·5	107	9·3	262	10·9
Ever breast-fed										
Yes	938	100	850	93·1	880	93·7	1267	96·6	3935	95·9
No	0	0	63	6·9	59	6·3	41	3·4	166	4·1
Age of weaning										
<6 months	41	4·4	62	6·8	80	8·5	168	12·8	351	8·6
≥6 months	897	95·6	851	93·2	859	91·5	1143	87·2	3750	91·4
Age of starting complementary foods										
<6 months	699	74·5	670	73·4	720	76·7	846	64·5	2935	71·6
≥6 months	239	25·5	243	26·6	219	23·3	465	36·5	1166	28·4
Child weight and height										
Weight (kg), mean and sd	12·53	0·06	12·79	0·06	12·89	0·06	13·32	0·05	12·88	0·04
Height (cm), mean and sd	91·06	0·21	91·77	0·21	91·76	0·18	92·79	0·16	91·81	0·13
BMI (kg/m^2^)†, mean and sd	15·07	0·04	15·15	0·05	15·24	0·04	15·42	0·04	15·22	0·27
Parental level										
Maternal										
Mother’s age										
<30 years	467	49·8	368	40·3	339	36·1	624	47·6	1798	43·8
≥30 years	471	50·2	545	59·7	600	63·9	687	52·4	2303	56·2
Mother’s BMI‡										
Underweight	111	11·8	95	10·4	75	8·0	121	9·2	402	9·8
Healthy weight	671	71·5	636	69·7	606	64·5	759	57·9	2672	65·2
Overweight/obese	156	16·7	182	19·9	258	27·5	431	32·9	1027	25·0
Mother’s height§										
Healthy height	417	44·5	417	45·7	438	46·7	714	54·5	1986	48·4
Short stature	521	55·5	496	54·3	501	53·3	597	45·5	2115	51·6
Check up during pregnancy										
Yes	821	87·5	545	59·7	653	69·5	1247	95·1	2899	70·7
No	117	12·5	368	40·3	286	30·5	64	4·9	1202	29·3
Paternal										
Father’s age										
<30 years	217	23·1	156	17·2	145	15·4	181	13·8	699	17·0
≥30 years	721	76·9	757	82·8	794	84·6	1130	86·2	3402	83·0
Father’s BMI‡										
Underweight	109	11·6	105	11·5	113	12·0	140	10·7	467	11·4
Healthy weight	714	76·1	715	78·3	665	70·8	905	69·0	2999	73·1
Overweight/obese	115	12·3	93	10·2	161	17·2	266	20·3	635	15·5
Father’s height§										
Healthy height	377	40·2	376	41·2	403	42·9	545	41·6	1870	45·6
Short stature	561	59·8	537	58·8	536	57·1	766	58·4	2231	54·4
Parents’ marital status										
Currently married	938	100	913	100	939	100	1303	99·4	4093	99·8
Formerly married	0	0	0	0	0	0	8	0·6	8	0·2
Household level										
Mother’s education										
No education	96	10·2	58	6·4	56	6·0	38	2·9	248	6·1
Primary school	508	54·2	516	56·5	448	47·7	395	30·1	1867	45·5
Junior and high school	297	31·7	285	31·2	331	35·3	577	44·0	1490	36·3
University or more	37	3·9	54	5·9	104	11·0	301	23·0	496	12·1
Father’s education										
No education	70	7·5	41	4·5	39	4·2	19	1·5	169	4·1
Primary school	462	49·3	459	50·3	411	43·8	262	20·0	1594	38·9
Junior and high school	337	35·9	342	37·5	357	38·0	282	21·5	1318	32·1
University or more	69	7·3	71	7·7	132	14·0	748	57·0	1020	24·9
Household’s wealth index										
Poor	446	47·6	801	87·7	404	43·0	582	44·4	2233	54·5
Average	141	15·0	69	7·6	192	20·5	269	20·5	671	16·4
Rich	351	37·4	43	4·7	343	36·5	460	35·1	1197	29·1
Community level										
Housing area										
Urban	435	46·4	413	45·3	429	45·7	696	53·1	1973	48·1
Rural	503	53·6	500	54·7	510	54·3	615	46·9	2128	51·9
Region										
Sumatra	248	26·4	209	22·9	206	21·9	322	24·6	985	24·0
Java	466	49·7	502	55·0	519	55·3	647	49·4	2134	52·0
Bali and Nusa Tenggara Barat	130	13·9	115	12·6	103	11·0	204	15·6	552	13·5
Kalimantan and Sulawesi	94	10·0	87	9·5	111	11·8	138	10·4	430	10·5

† Based upon the 2006 WHO Child Growth Standards for children <5 years^(^ ^23^ ^)^.

‡ Based upon the WHO BMI International Classification using general cut-off points^(^ ^26^ ^)^.

§ Height-for-age *Z*-score <−2^(^ ^27^ ^)^.

### Prevalence of and risk factors for concurrent stunting and overweight

Table 2 shows the prevalence of concurrent stunting and overweight as well as the associated risk factors. The prevalence indicates that children aged 2·0–2·9 years were significantly more likely to be stunted and overweight than those children aged 4·0–4·9 years. Children whose fathers had a healthy BMI, whose mothers and fathers were of short stature, whose mothers had a check-up during pregnancy, who were breast-fed for ≥6 months or who lived in rural areas had a higher prevalence of concurrent stunting and overweight.

**Table 2:** Prevalence of concurrent stunting and overweight among children aged 2·0–4·9 years (*n* 4101), Indonesian Family Life Survey

			OR					
	Stunting and overweight		Unadjusted			Adjusted†		
Variable	%	95% CI	OR	95% CI	*P*	OR	95% CI	*P*
Prevalence in each wave								
Wave 1 (1993)	6·4	5·0, 8·2	Ref.					
Wave 2 (1997)	6·8	5·3, 8·6	1·09	0·76, 1·59	0·609			
Wave 3 (2000)	5·2	4·0, 6·8	0·81	0·55, 1·20	0·309			
Wave 4 (2007)	7·2	6·0, 8·8	1·29	0·93, 1·80	0·124			
Child level								
Age								
2·0–2·9 years	10·2*	8·7, 11·9	Ref.			Ref.		
3·0–3·9 years	5·8	4·7, 7·2	0·51	0·39, 0·68	<0·001	0·57	0·41, 0·81	0·001
4·0–4·9 years	3·8	2·9, 4·9	0·34	0·25, 0·47	<0·001	0·44	0·30, 0·65	<0·001
Sex								
Male	7·0	6·0, 8·2	Ref.					
Female	6·0	5·0, 7·1	0·85	0·67, 1·08	0·186			
Birth weight (*n* 2420)								
<2·5 kg	7·2	4·1, 12·2	Ref.					
2·5–<4·0 kg	6·0	5·0, 7·1	0·93	0·50, 1·74	0·817			
≥4·0 kg	9·5	6·5, 13·7	1·39	0·67, 2·89	0·382			
Ever breast-fed								
Yes	7·1	6·3, 8·1	Ref.					
No	12·5	5·3, 26·7	2·45	1·00, 5·99	0·050			
Age of weaning								
<6 months	2·1*	0·9, 5·1	Ref.			Ref.		
≥6 months	7·0	6·1, 8·1	3·49	1·42, 8·61	0·007	2·98	1·20, 7·41	0·010
Age of starting complementary foods								
<6 months	6·7	5·7, 7·9	Ref.					
≥6 months	8·1	6·5, 10·0	1·22	0·91, 1·63	0·167			
Parental level								
Maternal								
Mother’s age								
<30 years	6·8	5·7, 8·0	Ref.					
≥30 years	6·3	5·3, 7·3	0·88	0·69, 1·12	0·303			
Mother’s BMI‡								
Underweight	6·2	4·2, 9·0	Ref.					
Normal	6·7	5·8, 7·7	1·06	0·63, 1·41	0·779			
Overweight/obese	6·1	4·8, 7·7	0·94	0·54, 1·33	0·475			
Mother’s height§								
Normal height	4·5*	3·7, 5·5	Ref.			Ref.		
Short stature	8·4	7·3, 9·6	1·84	1·42, 2·38	<0·001	1·66	1·22, 2·58	0·001
Check-up during pregnancy								
Yes	7·0*	6·4, 8·0	Ref.					
No	5·2	4·1, 6·1	0·71	0·53, 0·94	0·010			
Paternal								
Father’s age								
<30 years	7·4	5·6, 9·7	Ref.					
≥30 years	6·2	5·4, 7·2	0·77	0·57, 1·06	0·120			
Father’s BMI‡								
Underweight	5·3*	3·5, 8·0	Ref.					
Normal	7·4	6·4, 8·5	1·31	0·85, 2·02	0·219			
Overweight/obese	2·4	1·4, 4·2	0·41	0·21, 0·85	0·016			
Father’s height§								
Normal height	4·9*	3·9, 6·1	Ref.					
Short stature	7·5	6·4, 8·7	1·38	1·05, 1·83	0·020			
Parents’ marital status								
Currently married	6·5	5·8, 7·3	Ref.					
Formerly married	0			n/a				
Household level								
Mother’s education								
No education	7·7	4·9, 11·7	Ref.					
Primary school	7·2	6·1, 8·5	0·97	0·59, 1·60	0·917			
Junior and high school	5·4	4·3, 6·6	0·72	0·42, 1·22	0·229			
University or more	6·5	4·6, 9·0	0·84	0·66, 2·07	0·584			
Father’s education								
No education	7·1	4·1, 12·1	Ref.					
Primary school	8·8	7·5, 10·3	1·58	0·85, 2·96	0·152			
Junior and high school	3·9	3·0, 5·1	0·7	0·36, 1·37	0·298			
University or more	6·1	4·8, 7·7	0·97	0·55, 2·04	0·868			
Household’s wealth index								
Poor	7·0	6·0, 8·1	Ref					
Average	5·6	4·0, 7·7	0·81	0·56, 1·16	0·253			
Rich	6·0	4·7, 7·5	0·86	0·64, 1·14	0·290			
Community level								
Housing area								
Urban	4·7*	3·9, 5·7	Ref.			Ref.		
Rural	8·1	7·0, 9·4	1·79	1·38, 2·35	<0·001	1·66	1·19, 2·32	0·003
Region								
Sumatra	6·9	5·5, 8·7	Ref.					
Java	6·7	5·7, 7·8	0·98	0·71, 1·47	0·923			
Bali and Nusa Tenggara Barat	6·3	4·6, 8·7	0·89	0·49, 1·63	0·711			
Kalimantan and Sulawesi	4·9	3·2, 7·4	0·68	0·35, 1·32	0·256			

Ref., reference category; n/a, not applicable. *P value <0·003 (Bonferroni adjusted).

† Independent variables adjusted for are child-, parental- and household-, and community-level factors.

‡ Based upon the WHO BMI International Classification using general cut-off points^(^ ^26^ ^)^.

§ Height-for-age *Z*-score <−2^(^ ^27^ ^)^.

Univariate analysis indicated that, compared with 1993, the odds of being stunted and overweight increased by 29% in 2007. Children aged 3·0–3·9 and 4·0–4·9 years, and those with overweight/obese fathers were significantly less likely to be stunted and overweight. Children who were breast-fed after the age of 6 months were 3·49 times more likely to be stunted and overweight than children who were breast-fed for less than 6 months. Children whose fathers and mothers were of short stature were significantly more likely to be stunted and overweight.

After adjusting for potential confounders, the risk factors for stunted and overweight were: youngest age group (2·0–2·9 years), breast-fed after the age of 6 months, born to mothers who were classified as having short stature and living in rural areas. There was no collinearity found in the final model.

### Odds of stunted children being overweight

**Fig. 1:**
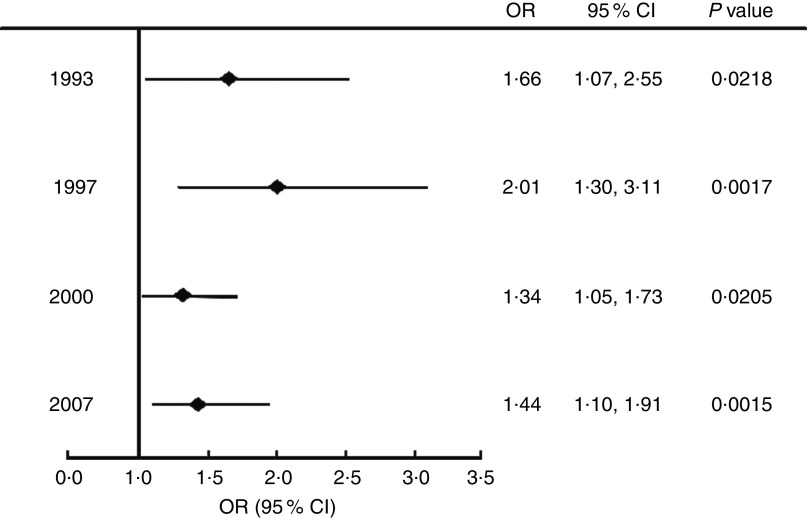
Odds ratios, with their 95% confidence intervals represented by horizontal bars, of stunted children aged 2·0–4·9 years being overweight; wave 1 (1993), wave 2 (1997), wave 3 (2000) and wave 4 (2007) of the Indonesian Family Life Survey

Figure 1 shows the odds of those who were stunted being overweight, compared with their healthy-height peers, for each wave of data collection. At all time points, stunted children were significantly more likely to be overweight than children who were not stunted (OR>1).

## Discussion

The current study presents a series of cross-sectional surveys from four different time points (1993, 1997, 2000 and 2007) over 14 years in Indonesia. We show that concurrent stunting and overweight occurs in the 2·0–4·9 year age group in Indonesian children, and is more likely in the 2·0–2·9 year age group, in children who were breast-fed for longer than 6 months, who lived in rural areas or whose mothers had short stature. In all four waves, stunted children were significantly more likely to be overweight/obese compared with children of healthy height. This is the first study to show the trends in the prevalence of concurrent stunting and overweight along with the associated risk factors in early childhood in a South-East Asian population of children.

The finding that children in the youngest age group (2·0–2·9 years) were more likely to experience concurrent stunting and overweight compared with the older children highlights the importance of interventions starting as early as possible. The WHO, in its policy briefs for stunting^(^ ^30^ ^)^ and also for overweight^(^ ^31^ ^)^, emphasizes the need for multisectoral approaches as well as interventions needing to occur prior to, during and beyond pregnancy.

As a country undergoing transition, an investment in education is definitely on the agenda in Indonesia. Over the duration of the study, the education level of both mothers and fathers improved, as shown by the decline in the percentage of mothers and fathers who had no formal education. Between waves 2 and 3, the number of parents who went to university nearly doubled. The same phenomenon happened between waves 3 and 4 for women. In many Asian cultures, when it comes to education, parents often prefer to send their boys to school, because they will become the head of the family. Within each wave in our study, there was a higher proportion of men with university education compared with women.

The present analysis showed no association between parental education and concurrent stunting and overweight. However, in our first paper^(^ ^17^ ^)^ we found that stunting itself was associated with mothers not having formal education. Our first paper also detailed the association between child overweight/obesity status and fathers who attended university^(^ ^17^ ^)^, a finding in keeping with other studies that have shown a positive association between socio-economic position and child obesity in low- and middle-income countries^(^ ^32^ ^,^ ^33^ ^)^.

Although several reports have shown that the double burden of malnutrition may occur in the same family or even individual in Indonesia^(^ ^1^ ^,^ ^7^ ^,^ ^34^ ^–^ ^36^ ^)^, none has specifically looked at the prevalence of this phenomenon in very young children or explored the associated risk factors. Our findings show the prevalence of concurrent stunting and overweight in Indonesia to be more than 5% in all four waves, with an overall rate increase of 0·06% per year over the 14-year period. Although this prevalence is still relatively low, studies from other countries have generally shown a lower prevalence of combined stunting and overweight in both school-aged and pre-school-aged children^(^ ^4^ ^,^ ^10^ ^,^ ^12^ ^,^ ^14^ ^,^ ^15^ ^,^ ^37^ ^,^ ^38^ ^)^. The two exceptions are a report from Uruguay, where a concurrent prevalence of 13·2% was documented in 2046 children aged 0–59 months^(^ ^13^ ^)^, and a South African report^(^ ^16^ ^)^ which showed a prevalence of 19%, albeit in a small sample of 162 children aged 3 years. Another important factor is that different definitions and cut-off points are used to determine the prevalence of the double burden of malnutrition. The lack of one standardised definition and set of cut-offs makes interpretation of different studies more difficult. Future research in this area would benefit from a consensus on standard definitions and cut-off points. Other research recommendations include more focus on eating behaviours and physical activity. There is also a need for prospectively designed studies.

There are few reports of risk factors associated with concurrent stunting and overweight in childhood, particularly in nationally representative samples^(^ ^9^ ^,^ ^39^ ^)^. In a study of 7555 children aged 2–6 years from rural areas in Mexico, Fernald and Neufeld found similar results to our study, whereby concurrent stunting and overweight was higher in children whose mothers had short stature^(^ ^9^ ^)^, a finding also documented by Keino *et al*. in their review on determinants of this phenomenon in children from sub-Saharan Africa^(^ ^39^ ^)^. Other factors found to be related to concurrent stunting from these studies were lower socio-economic status, lower maternal education, large household size and younger mothers^(^ ^9^ ^,^ ^39^ ^)^. One of the findings in our study is the apparently counterintuitive observation that a longer duration of breast-feeding is associated with concurrent stunting and overweight. Several studies have shown that prolonged breast-feeding duration protects against obesity in childhood^(^ ^40^ ^,^ ^41^ ^)^ and also stunting^(^ ^42^ ^,^ ^43^ ^)^. Therefore, our finding must be interpreted with caution, keeping in mind that many other factors influence a child’s nutritional status, including the age of commencing complementary feeding, the type of complementary feeding, the use of formula milk and the family hygiene and sanitation conditions.

We compared the four risk factors associated with concurrent stunting and overweight found in the current analysis with those we identified for stunting or for overweight as individual phenomena in our previous paper^(^ ^17^ ^)^. One – being in the youngest age group (2·0–2·9 years) – was a similar risk factor for being overweight alone. The other associated risk factors – being breast-fed for more than 6 months, having a short-statured mother and living in a rural area – were similar to the risk factors for stunting alone. No new risk factors associated with concurrent stunting and overweight were identified. However, as we have earlier stated in the ‘Methods’ section, we can only report on the potential risk factors available from the data sets.

We also investigated whether stunted children are at greater risk of being overweight compared with their healthy-height peers. Bove *et al*. had similar results to our findings, where stunted children aged 0–4·9 years were 2·7 (95% CI 1·8, 4·1) times more likely to be overweight/obese (BMI *Z*-score >+2) than children of healthy height^(^ ^13^ ^)^. A study by Popkin *et al*. also showed in children aged 3 to 9 years from four different countries (Russia, China, South Africa and Brazil) that there was a significant association between stunting and overweight/obesity (OR of 1·7 to 7·7)^(^ ^11^ ^)^. Interestingly, in a 2004 study from Indonesia – among 3010 prepubertal school-aged children, randomly sampled from one urban (Yogyakarta) and one rural area (Gunung Kidul) – the stunted children were less likely to be overweight compared with their non-stunted peers^(^ ^36^ ^)^.

Stunting is closely related to lower cognitive performance^(^ ^1^ ^,^ ^44^ ^,^ ^45^ ^)^, poorer motor development^(^ ^1^ ^,^ ^45^ ^)^ and lower immune function^(^ ^1^ ^)^. Overweight is an important underlying cause for the occurrence of many non-communicable diseases^(^ ^46^ ^)^, one of the main causes of death and disability both in Indonesia and globally^(^ ^35^ ^,^ ^47^ ^)^. Thus, the consequences of concurrent stunting and overweight, although never having been addressed previously, are of great importance. Furthermore, as has been highlighted in reports from the World Bank and other reviews^(^ ^1^ ^,^ ^8^ ^,^ ^34^ ^)^, there are internationally recognised policies and strategies for combating stunting^(^ ^1^ ^,^ ^8^ ^,^ ^30^ ^,^ ^48^ ^,^ ^49^ ^)^ and overweight/obesity^(^ ^1^ ^,^ ^8^ ^,^ ^31^ ^)^; although these have usually been addressed separately. A few very recent policies have specifically addressed the double burden of malnutrition, such as with the South African food-based dietary guidelines^(^ ^50^ ^)^.

The strengths of our study include the large sample size of participants and the use of trained observers to undertake anthropometric measurements. The representative nature of sampling and the use of similar methods throughout the four waves allow comparison of results between waves. We also included sampling weights during the analyses to reduce potential bias.

One limitation of the study is the cross-sectional design, limiting the ability to explore causation. Another is the unavailability, or limited amount, of data on children’s and adults’ physical activity. In addition, we were not able to assess other aspects of the child’s eating behaviours (including their breast-feeding details: exclusively, combined with formula milk, or predominantly formula milk), nor the family hygiene and sanitation conditions, all of which influence children’s nutritional status. Even though 95% of these children were ever breast-fed, more than 70% were started on complementary foods at less than 6 months of age. The quality of these complementary foods will also have an impact on weight status and linear growth.

## Conclusion

In conclusion, our paper demonstrates that the double burden of malnutrition occurs at the individual level in Indonesian children aged 2·0–4·9 years. Such data should serve as the catalyst for developing policy and programmes in dealing with concurrent stunting and overweight. It is important that both policy makers and health practitioners work together in addressing this public health problem.
